# The effect of hard tissue surgical changes on soft tissue displacement: a pilot CBCT study

**DOI:** 10.1590/2177-6709.22.5.039-046.oar

**Published:** 2017

**Authors:** Leonardo Koerich, Daniel Paludo Brunetto, Eduardo Terumi Blatt Ohira

**Affiliations:** 1 Virginia Commonwealth University, International Dental Program (Richmond, USA).; 2 Universidade Federal do Paraná (Curitiba/PR, Brazil).; 3 Private practice (Pomerode/SC, Brazil).

**Keywords:** Cone-Beam CT, Three-dimensional image, Orthognathic surgery

## Abstract

**Introduction:**

This pilot study had as main objective to test the reliability of a new method to evaluate orthognathic surgery outcomes and also, to understand the effect of hard tissue changes on soft tissue displacement.

**Methods:**

The sample consisted of eight patients that underwent bimaxillary advancement and had CBCT at two time points (before surgery and 6-8 months follow-up). Voxel-based cranial base superimposition was used to register the scans. A different technique of iterative closest point (ICP) was used to measure and correlate the changes. The average displacement of 15 areas (4 hard tissue and 11 soft tissue) were measured twice.

**Results:**

ICC was > 0.99 for all areas. Changes in the tip of the nose did not correlate with changes in any maxillary area, whereas soft tissue A point, A point and upper lips had correlation with several areas. The highest correlation for the maxilla was between the upper lip and the left/right supra cheilion (*p*< 0.001, *r*= 0.91 and *p*< 0.001, *r*= 0.93, respectively). In the mandible, the majority of the correlations involved soft tissue pogonion, pogonion and lower incisors, with the strongest one between pogonion and lower incisors (*p*< 0.001, *r*= 0.98).

**Conclusion:**

With the proper case selection, ICP is a reliable method that can be used to assess three-dimensional changes.

## RESUMO

**Objetivo:**

o objetivo deste estudo piloto foi testar a confiabilidade de um novo método para avaliar os resultados da cirurgia ortognática e entender o efeito das alterações dos tecidos duros no deslocamento dos tecidos moles.

**Métodos:**

a amostra selecionada consistiu de oito pacientes submetidos a avanço bimaxilar que possuíam TCFC em dois períodos de tempo (antes da cirurgia e de 6 a 8 meses após a cirurgia). A sobreposição da base do crânio pelo método *voxel-based* foi utilizada para registrar as varreduras, enquanto uma técnica diferente, de ponto iterativo mais próximo (ICP), foi usada para medir e correlacionar as mudanças. O deslocamento médio de 15 áreas (4 em tecidos duros e 11 em tecidos moles) foi mensurado duas vezes.

**Resultados:**

o ICC foi > 0,99 para todas as áreas. As alterações da ponta do nariz não se correlacionaram com alterações em qualquer área da maxila, enquanto o ponto A em tecido mole, o ponto A e os lábios superiores apresentaram correlação com várias áreas. A maior correlação para a maxila foi entre o lábio superior e a supracomissura esquerda/direita (*p*< 0,001, *r* = 0,91 e *p*< 0,001, *r* = 0,93, respectivamente). Na mandíbula, a maioria das correlações envolveu o pogônio em tecido mole, pogônio e incisivos inferiores, sendo a maior entre pogônio e incisivos inferiores (*p*< 0,001, *r*= 0,98).

**Conclusão:**

com a seleção adequada do caso, o ICP é um método confiável que pode ser utilizado para avaliar mudanças tridimensionais.

## INTRODUCTION

Orthodontic treatment aims to achieve a stable occlusion and improve facial esthetics. For some patients, the malocclusion is caused by a severe jaw discrepancy that cannot be compensated only by tooth movement. These patients would benefit greatly from orthodontic-surgical treatment, which will correct both the jaw position and corresponding overlying soft tissue.^1^

To assess the soft and hard tissue, lateral cephalometric radiography has been extensively used and studied.^2^ ^,^ ^3^ With pre- and post-treatment cephalometric superimposition techniques,^4^ it is possible to assess treatment outcomes. However, there are several limitations, including the lack of a three-dimensional (3D) assessment of the patient when assessing only from a sagittal view. 

Cone-beam computed tomography (CBCT) is a great imaging modality to provide 3D visualization of structures. Despite the fact that there is radiation exposure and the acquisition time is longer - when compared to laser scans (7.5s)^5^ and stereophotogrammetry (1.5ms, at highest resolution) -,^6^ CBCT has the benefit of providing a combined soft and hard tissue view. Additionally, 3D soft tissue images alone have no stable structure to be used for superimposition of pre- and post-surgical images, for example, as compared to the CBCT voxel-wise cranial base superimposition.^7^ Usually, registration is done in areas not modified by the surgery, such as the forehead.^8^ ^,^ ^9^ However, this area can be affected due to weight change over time and facial expressions during image acquisition.

Although cephalometric radiography has several limitations, most of the studies were conducted using similar methods over the years, which included acquisition of two or more cephalometric radiographs, cephalometric tracing and superimposition of different time points.^10^ ^-^ ^12^ In contrast, there is no consensus among studies regarding the method for making 3D cephalometric measurements or evaluating changes using superimposition. McCance et al.^13^ used medical CT to evaluate hard and soft tissue changes after orthognathic surgery. Despite the limitation of the superimposition method used (landmark-based), it was an innovative study and likely the first to attempt this type of analysis. Kim et al.^14^ proposed a method without cranial base superimposition, using a grid to evaluate different areas of the face. With variations in methodology, other authors^15^ ^-^ ^19^ used similar techniques to evaluate soft tissue changes: cranial base superimposition and measuring the displacement of landmarks or iterative closest point (ICP).

In order to more accurately evaluate 3D hard and soft tissue changes, a new method for measurements was assessed using areas rather than landmarks as references. The aim of this pilot study was to evaluate: 1) the reliability of changes measured in areas and, 2) correlations between hard and soft tissue changes. 

## MATERIAL AND METHODS

This research was conducted after ethical approval of the Institute of Collective Health Studies committee from the Federal University of Rio de Janeiro (#115/2011). This was a retrospective study and the sample was collected from a private practice.

The inclusion criteria were patients with: 1) bimaxillary advancement surgery for either esthetic or functional reasons; 2) pre- and post-operative scans taken with the same CBCT machine; 3) surgical pre-treatment age between 18 and 30 years old (mean = 24.1 ± 2.39 years). Exclusion criteria were 1) significant maxillary and/or mandibular transverse skeletal asymmetry (> 3 mm); 2) concomitant genioplasty; 3) syndromes. A total of 8 patients were selected (5 males and 3 females).

CBCT scans (i-Cat, Imaging Sciences International, Hatfield, PA) were taken two to three weeks prior to the surgery (T_1_) and six to eight months after surgery (T_2_). The same technician operated the machine for all scans using the following protocol: 120kV, 5mA, 13x17 cm field of view, 0.4mm voxel size and 20 seconds for image acquisition. Patients were oriented to keep teeth in gentle contact and to breathe steadily during scan time. 

The surgery was performed by the same surgical team and the surgical technique was the same for all patients: Le Fort 1 osteotomy in the maxilla and bilateral sagittal split osteotomy in the mandible. Rigid internal fixation with plates and screws was used. The suturing technique used for the maxilla was VY closure.

**Figure 1:**
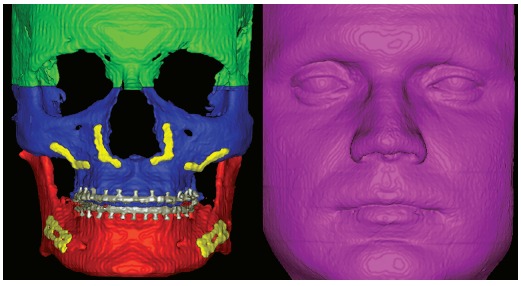
Segmentation of the regions of interest. Three regions were exported as .STL files: maxilla and upper teeth, mandible and lower teeth, and soft tissue.

Image analysis partially followed the protocol suggested by Cevidanes et al.^7^ The CBCT’s had the voxel size reformatted from 0.4mm to 0.5mm and the cranial base superimposition was performed using the voxel-based method with the software Imagine (National Institutes of Health, USA). The superimposition used T_1_ as a reference and moved T_2_ with six degrees of freedom (translation and rotation for x, y and z). The maxilla, mandible and soft tissue were segmented separately using the software ITK-Snap.^20^ The segmentation technique consisted of different labels for each of the three structures (Fig. 1). 

**Figure 2:**
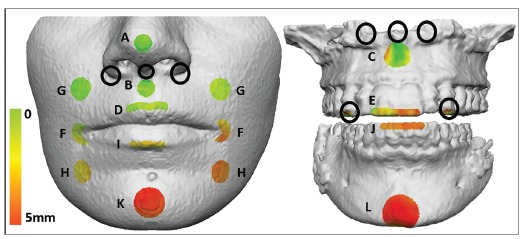
Soft and hard tissue areas with color-coded distances in one patient. A) Nasal tip, B) Soft A Point, C) A Point, D) Upper Lip, E) Upper Incisors, F) Left/Right Cheilion, G) Left/Right Supra Cheilion, H) Left/Right Sub Cheilion, I) Lower Lip, J) Lower Incisors, K) Soft Pogonion, L) Pogonion. Black circles are the areas initially evaluated but removed because they were not reliable.

Three different .STL files (maxilla, mandible and soft tissue) for each time point were exported from ITK-Snap and imported into VAM (Canfield Scientific, Fairfield, NJ) for the 3D analyses. The iterative closest point (ICP) technique was used to assess the changes between T_1_ and T_2_. The method consists of finding the closest points in specific areas between T_1_ and T_2_. Using the “surface paint area” tool provided by the software and after several screening tests, one operator selected a total of 4 areas on the hard tissue and 11 on the soft tissue (Fig. 2). The software provided three values for each area: minimal, maximal and average displacement. For this study, only the average displacement for each area was selected. 

### Statistical analysis 

To measure the reproducibility of the method, all areas were re-measured by the same operator after one-month washout period and the intraclass correlation coefficient (ICC) was calculated followed by statistical comparison. Due to the small sample size, Spearman rank correlation was used to correlate the hard and soft tissue changes. Correlation analyses were divided into maxillary and mandibular areas and significance was set at *p*< 0.05.

## RESULTS

ICC results are depicted in Table 1 and were considered excellent ( > 0.99) for all measurements. Table 2 shows descriptive statistics for the displacement in each area. Mean maxillary displacement was 3.14 ± 1.26 mm (measured at the area of A Point) and mandibular displacement was 9.08 ± 3.01 mm (measured at the area of Pogonion). Table 3 shows the Spearman correlations for the maxilla. Movement of the nasal tip did not correlate to movement at any other area. Soft tissue A point movement correlated with every structure except right cheilion (*p*= 0.025) and tip of the nose (*p*= 0.041). Hard tissue A point movement correlated with movement of soft tissue A point (*p*= 0.04), upper lip (*p*< 0.001), left/right supra cheilion (*p*= 0.01 and 0.01) and left cheilion (*p*= 0.00). Upper lip also correlated with upper incisors (*p*= 0.04), left/right supra cheilion (*p*= 0.00 and 0.00) and left cheilion (*p*= 0.01). Table 4 shows the Spearman correlations for the mandible. There was correlation between movement of pogonion and the lower incisors (*p*= 0.00), pogonion and soft tissue pogonion (*p*= 0.04) and soft tissue pogonion and the lower incisors (*p*= 0.02).The lower lip correlated with right sub cheilion (*p*= 0.04), and soft tissue pogonion correlated with left sub cheilion (*p*= 0.02).

**Table 1:** Intra-class correlation coefficient (ICC) showing reproducibility of the measurements and the mean difference between measurements at T_1_ and T_2_.

	ICC	Differences (mm)				
		Min	Max	Mean	SD	95% CI
Nasal Tip	0.999	-0.03	0.06	0.02	0.04	-0.01 to 0.04
Soft A Point	0.997	-0.11	0.23	0.06	0.10	0.00 to 0.13
A Point	0.995	-0.13	0.18	-0.01	0.11	-0.08 to 0.07
Upper Lip	0.999	-0.07	0.13	-0.01	0.06	-0.04 to 0.05
Upper Incisors	0.999	-0.09	0.10	0.01	0.06	-0.03 to 0.05
Left Cheilion	0.999	-0.17	0.07	-0.05	0.07	-0.10 to 0.00
Right Cheilion	0.997	-0.07	0.24	0.02	0.11	-0.04 to 0.09
Left Supra Cheilion	0.999	-0.11	0.17	-0.01	0.08	-0.06 to 0.05
Right Supra Cheilion	0.998	-0.10	0.12	-0.01	0.07	-0.05 to 0.03
Left Sub Cheilion	0.997	-0.18	0.21	-0.02	0.12	-0.09 to 0.06
Right Sub Cheilion	0.996	-0.23	0.08	-0.05	0.10	-0.12 to 0.02
Lower Lip	0.999	-0.10	0.17	0.00	0.09	-0.05 to 0.06
Lower Incisors	0.999	-0.01	0.17	0.07	0.07	0.03 to 0.12
Soft Pogonion	1.000	-0.10	0.06	0.00	0.05	-0.03 to 0.03
Pogonion	1.000	-0.06	0.04	0.01	0.04	-0.02 to 0.03

Min = minimum; Max = maximum; SD = standard deviation; CI = confidence interval.

**Table 2:** Descriptive statistics showing the displacement (mm) for each area.

	Min	Max	Mean	SD
Nasal Tip	0.52	2.90	1.51	0.72
Soft A Point	0.63	3.46	1.88	1.16
A Point	1.27	5.05	3.14	1.26
Upper Lip	1.52	5.74	3.36	1.51
Upper Incisors	0.53	4.21	2.01	1.18
Left Cheilion	1.51	6.01	4.13	1.29
Right Cheilion	2.28	6.02	4.08	1.40
Left Supra Cheilion	2.80	6.59	4.29	1.58
Right Supra Cheilion	1.87	4.94	3.49	1.29
Left Sub Cheilion	4.69	8.51	5.86	1.47
Right Sub Cheilion	3.80	7.03	5.54	1.18
Lower Lip	3.57	12.25	6.48	2.71
Lower Incisors	4.98	10.89	7.38	1.84
Soft Pogonion	6.04	14.65	8.91	2.62
Pogonion	6.08	15.72	9.08	3.01

Min = minimum; Max = maximum; SD = standard deviation.

**Table 3:** Spearman correlation between structures in the upper jaw.

	Nasal Tip	Soft A Point	A Point	Upper Lip	Upper Incisors	Left Cheilion	Right Cheilion	Left Supra Cheilion	Right Supra Cheilion
Nasal Tip	-	0.41	0.16	0.48	0.25	0.09	0.25	0.48	0.37
Soft A Point	-0.10	-	0.04	< 0.01	0.03	0.04	0.25	0.01	0.03
A Point	0.40	0.66	-	< 0.01	0.08	< 0.01	0.08	0.01	0.01
Upper Lip	0.02	0.86	0.86	-	0.04	0.01	0.07	< 0.01	< 0.01
Upper Incisors	0.29	0.68	0.55	0.64	-	0.04	0.33	0.08	0.13
Left Cheilion	0.52	0.66	0.86	0.83	0.67	-	0.25	0.03	0.04
Right Cheilion	-0.29	0.29	0.55	0.57	-0.19	0.29	-	0.05	0.02
Left Supra Cheilion	-0.02	0.79	0.76	0.91	0.55	0.69	0.62	-	0.01
Right Supra Cheilion	-0.14	0.67	0.79	0.93	0.45	0.67	0.74	0.83	-

Correlations are below and p values above the dashes.

Statistically significant values (*p*< 0.05) are shown in bold.

**Table 4:** Spearman correlation between structures in the lower jaw.

	Left Sub Cheilion	Right Sub Cheilion	Lower Lip	Lower Incisors	Soft Pogonion	Pogonion
Left Sub Cheilion	-	0.21	0.37	0.19	0.02	0.16
Right Sub Cheilion	0.33	-	0.04	0.09	0.08	0.08
Lower Lip	0.14	0.67	-	0.10	0.12	0.09
Lower Incisors	0.36	0.52	0.50	-	0.02	<0.01
Soft Pogonion	0.74	0.55	0.48	0.71	-	0.04
Pogonion	0.40	0.55	0.52	0.98	0.67	-

Correlations are below and p values above the dashes.

Statistically significant values (p<0.05) are shown in bold.

## DISCUSSION

The main innovation brought about by this study was an increase in the area being evaluated, when compared to other 3D techniques. Besides that, the focus was on the average displacement rather than maximum displacement only. Initial analysis included other bony areas to be evaluated, such as anterior nasal spine and adjacent areas, and the upper canines (Fig. 2). They were excluded because either there was an osteotomy done in the area creating bony defects or there were artifacts. Regarding soft tissue, a subnasal and two subalar (one at each side of the nose) areas that were initially used, were removed after the screening tests. Due to their specific location in the curvature of the nasal base, they frequently have inward and outward (negative and positive values) changes, potentially underestimating the final result. That is the reason why cheilion points had to follow the outline contour of the lips instead of being a circle, to avoid the negative values on the lip corners (Fig. 2). The measurements for the upper and lower teeth were restricted to the incisal third because of orthodontic bracket artifacts. Nevertheless, beam-hardening effects remained a limitation, possibly affecting the measurements in the area around the teeth.

Another important aspect of the methodology was the segmentation with different labels. ICP detected the closest point between T_1_ and T_2_. In other words, if the segmentations were with the same label, the software could compare the distances between the lip in T_2_ and the teeth in T_1_. The software could also compare the distance between the upper incisors in T_2_ and lower incisors in T_1_ if the jaws were segmented with the same label. The differentiation between soft tissue, maxilla and mandible demanded more time but decreased the chance of errors. 

The reproducibility of the measurements was very high. All fifteen measurements had an ICC higher than 0.99 and, for two of them, it was equal to 1. The highest difference between all the measurements was smaller than 0.25mm and 95% of the cases had differences smaller than 0.13 mm (Table 1). This shows that it was possible to obtain measurements using larger areas for evaluation without having to break down the distances in different 2D vectors.

In the maxilla, there was no significant correlation between movement of the tip of the nose and any other area. Evaluating Class III patients that underwent maxillary advancement associated with mandibular setback, Baik and Kim^21^ did not find correlation between tip of the nose and hard tissue changes. Soncul and Bamber^22^ found that patients that had undergone maxillary impaction and advancement had soft tissue changes of the tip of the nose corresponding to 29% of the bony change. Interestingly, soft tissue A point movement was significantly correlated with changes in bilateral structures such as left/right supra cheilion and left cheilion. Upper lip changes also correlated with left/right supra cheilion and both supra cheilion areas correlated to each other. In a study that assessed bilateral soft tissue structures, Oh et al^16^ evaluated changes after two to six months from surgery and found that both supra cheilion areas significantly correlated to each other (0.79) and the same was true for to left and right cheilion (0.78). In the current study the supra cheilion area movements correlated to each other (0.83). However, the right and left cheilion areas did not correlate to each other (0.29). The upper lip and upper incisors showed moderate correlation (0.64). However, the following factors may have contributed to this outcome: braces influencing the upper lip projection;^23^ metal brackets generating artifacts affecting T_1_ segmentation; and tooth movement (torque) during the finishing orthodontic stage that might have affected the measurements. 

Lower lip movement had a weak correlation with the lower incisors in the current study (0.50). Almeida et al.^19^ found similar results (0.55) for these variables in a one-year follow up. In the same study, pogonion and soft tissue pogonion movements were strongly correlated (0.86) while in this study, the correlation was slightly lower (0.67). Different than supra cheilion results, the sub cheilion areas did not correlate to each other. Two possible explanations are 1) significant difference between maxillary and mandibular advancement in the current sample and/or 2) a small rotation during the mandibular advancement that could have contributed to differences between the left and right side. Baik and Kim^21^ mentioned that, due to the semicircular anatomy of the maxilla and the mandible, changes in soft tissue are smaller when the distance to the midline increases. In the current study, the descriptive analysis showed that subcheilion had smaller advancements when compared to soft tissue pogonion; however, supra cheilion displayed greater movement than soft tissue A point.

**Figure 3:**
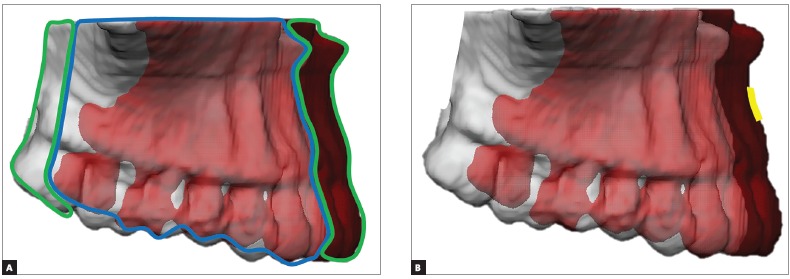
Simulation of maxillary advancement (5 mm) comparing two types of measurements. A) Shows how the mean displacement is measured if 100% of the area is selected. Areas inside the green contour (anterior and posterior) are expected to have the highest displacement because there is no overlap between T_1_ and T_2_. The areas inside the blue contour (between anterior and posterior) are expected to have the smallest displacement because T_1_ and T_2_ are overlapping. Since the vast majority of the area is inside the blue contour, when measuring 100% of the distance between T_1_ and T_2_, the mean displacement is grossly underestimated. B) Shows how the present method works: measuring only the area of interest (yellow) where the displacement was more significant.

Jabar et al.^24^ suggested that the ICP method grossly underestimates changes in the maxilla and the mandible, based on their study using simulated orthognathic surgery. However, there is a limitation in that study that needs to be evaluated carefully. First, the authors used 90% and 100% of the mesh area to make the evaluation. When the maxilla is advanced, the majority of the T_2_ surface overlaps on the T_1_ surface (Fig. 3).Therefore, the displacement values in the overlapped area are expected to be zero or very close to zero, bringing the mean average displacement value down and underestimating the final result. That is the main reason why studies^19^ ^,^ ^25^ ^,^ ^26^ select specific areas (anterior part of the maxilla or mandible) to make the ICP evaluation. In that situation, less than 1% of the area was used to measure (only the maximum displacement) and the area had no overlap. Ultimately, small regions were selected to prevent inclusion of plates, screws, bony defects and non-interest areas, in an effort to obtain a more significant amount of “affected” area. In addition, the size of the area selected has a direct effect on the amount of displacement. As the area size increases, the average displacement tends to decrease.

The biggest challenge to obtain accurate results with ICP is case selection. Based on the results of this study and the limitations presented by Jabar et al,^24^ the following are appropriate procedures for inclusion: maxillary advancement, mandibular advancement or setback, and bimaxillary advancement (when the movements are mainly sagittal). It is important to know that the amount of pitch and yaw rotation and even moderate vertical changes are important limiting factors for this method. This method could likely be used for non-growing orthodontic patients that have extractions done followed by retraction of incisors, or retraction with mini-plates. It is understood that this may reduce the external validity of the study and the method, yet it is preferable to have more accurate data for a select group of patients than to incur errors when extrapolating data for inappropriate procedures.

## CONCLUSION

This study showed a new technique for investigating hard and soft tissue changes and the relationship between them. Changes in areas can be measured in 3D with high reproducibility. The correlations found in this and future studies with more subjects can help to predict soft tissue outcomes of orthodontic-surgical therapies and aid professionals in developing goal-oriented treatment plans.
